# Skin, Liver, and Kidney Interactions Contribute to Skin Dryness in Aging KK-Ay/Tajcl Mice

**DOI:** 10.3390/biomedicines10102648

**Published:** 2022-10-20

**Authors:** Keiichi Hiramoto, Kenji Goto, Shota Tanaka, Tsuneki Horikawa, Kazuya Ooi

**Affiliations:** 1 Department of Pharmaceutical Sciences, Suzuka University of Medical Science, Suzuka 513-8670, Japan; 2 Research Laboratories, Nichinichi Pharmaceutical Co., Ltd., Iga 518-1417, Japan

**Keywords:** type 2 diabetes, collagen, mast cell, tumor necrosis factor-α, interleukin-6, reactive oxygen species

## Abstract

Type 2 diabetes is a lifestyle-related disease that affects people worldwide and is especially prevalent in the elderly. Many elderly people with diabetes also complain of dry skin; however, the relationship between aging and dry skin in type 2 diabetes is unknown. The purpose of this study was to examine the interaction between aging and dry skin using the specific pathogen-free KK-Ay/TaJcl type 2 diabetes mouse model. Skin dryness in this model increases with age and was evaluated at 10, 27, 40, and 50 weeks. We observed increased mast cell expression, increased histamine and matrix metalloproteinase-1 levels, and decreased collagen expression in the skin of aging KK-Ay/TaJcl mice. In addition, the increased expression of angiopoietin 2, interleukin-6, tumor necrosis factor-α, and endostatin in the blood indicated kidney damage in this model. Aging KK-Ay/TaJcl mice also showed fatty liver pathology, which led to increased reactive oxygen species in the blood and liver, as well as the increased expression of M1 macrophages in the liver. These results showed that dry skin is associated with skin, kidney, and liver interactions in an aging type 2 diabetes mouse model.

## 1. Introduction

Diabetes can manifest as type 1 and type 2 diabetes. Type 2 diabetes is caused by a decrease in insulin secretion and abnormal action due to lifestyle changes and accounts for more than 90% of all patients with diabetes. Type 2 diabetes often develops after the age of 40, with the number of cases increasing with age [1,2,3]. Underlying conditions of diabetes includes chronic hyperglycemia, which damages blood vessels and affects blood flow to the eyes, kidneys, and nerves, causing nephropathy, retinopathy, and neuropathy. Diabetes also causes polydipsia, pollakiuria, fatigue, and pruritus. Of these diseases, the complication rate of diabetes and pruritus is less than 10% [4,5]. Pruritus not only lowers patient QOL, but also causes problematic infections. Thus, diabetes is associated with systemic or localized pruritus dermatitis, suggesting dry skin is involved in pruritus dermatitis [4,6].

We previously examined dry skin using the KK-Ay/Tajcl type 2 diabetes mouse model and reported a marked increase in histamine in these mice [7]. In addition, a decrease in hyaluronic acid was observed, which was caused by inflammatory cytokines interleukin (IL)-6 and tumor necrosis factor (TNF)-α that are secreted from macrophages induced in adipocytes due to obesity [7].

Skin dryness and pruritus also involve the kidneys; diabetes can cause kidney abnormalities and reduce renal function [8]. Furthermore, the decrease in renal function can cause the accumulation of waste products in the blood and cause skin itching though the stimulation of the *n*-opioid receptor [9]. However, the detailed mechanism of how kidney damage is associated with dry skin is unknown. Additionally, in type 2 diabetes, insulin resistance promotes triglyceride synthesis in the liver, which is accompanied by fatty liver development [10]. Adipocytes release inflammatory cytokines that activate mast cells and can lead to dry skin [11], and thus, the dryness of the skin is related to both the kidneys and liver in type 2 diabetes. Moreover, these symptoms can become more severe as type 2 diabetes develops with age.

In this study, we used the KK-Ay/Tajcl type 2 diabetes mouse model to observe the skin, kidneys, and liver over time to examine the degree of their association during aging.

## 2. Materials and Methods

### 2.1. Animal Experiments

Specific-pathogen-free (SPF) C57BL/6N and KK-Ay/TaJcl male mice (CLEA Japan Inc., Tokyo, Japan) were used in the study at 10-, 27-, 40-, and 50-w-old (*n* = 5). Mice were individually maintained in cages in an air-conditioned room at 23 ± 1 °C under SPF conditions with a 12 h light/12 h dark cycle, with free access to drinking water and a pelleted basal diet. Each week, body weight (g), kidney weight (g), and liver weight (g) were measured. Additionally, small blood samples were collected from the tail vein of each mouse to measure blood glucose levels. Mice with a blood glucose level of 300 mg/dL or higher were considered to be diabetic. The study was performed in strict accordance with the recommendations of the Guide for the Care and Use of Laboratory Animals of Suzuka University of Medical Science (Approval number: 34). All surgeries were performed under pentobarbital anesthesia, and efforts were made to minimize animal suffering.

### 2.2. Measurement of Dorsal Skin Transept Water Loss and Capacitance

After anesthesia induction, transept water loss (TEWL) and the skin hydration levels of the dorsal skin were measured 4 w after initiating the experiment, as previously described [12]. Briefly, TEWL is a permeability measurement that reflects the barrier function of the skin that was determined using a Tewameter TM300 (Courage + Khazaka Electronic GmbH, Cologne, Germany). Skin hydration levels were assessed 3 times using a Corneometer CM825 (Courage + Khazaka Electronic GmbH). The degree of skin hydration was determined by measuring electrical capacitance in arbitrary units. For this measurement, the same pressure and the skin region were used for each mouse.

### 2.3. Preparation and Staining of Dorsal Skin, Kidney, and Liver Samples

Mice were sacrificed at each determined end point, and dorsal skin, kidney, and liver specimens were fixed at 4 °C in 4% phosphate-buffered paraformaldehyde, and then transferred into glucose (20% in PBS), embedded in frozen Tissue Tek OCT compound (Sakura Finetek, Co., Ltd. Tokyo, Japan), and cut into 5 μm-thick sections. These sections were stained with hematoxylin–eosin using established procedures to enable the histological analysis of the skin. To evaluate collagen expression, samples were stained using the Masson trichrome technique (modified Masson’s trichrome stain kit; ScyTec Laboratories, Inc., Logan, UT, USA) [13]. Additionally, skin specimens were stained with toluidine blue to visualize mast cells. The stained skin tissues were microscopically evaluated according to conventional procedures. BX51 Olympus microscope with UPlanSApo (Olympus, Tokyo, Japan) was used for these histological analyses.

### 2.4. Enzyme-Linked Immunosorbent Assay (ELISA) Analysis

Blood was collected from the hearts of mice at the determined end points and centrifuged at 3000 rpm to isolate the plasma by a microvolume high-speed cooling centrifuge MX-201 (TOMY, Tokyo, Japan) and stored at −20 °C. The dorsal skin, livers, and kidneys were excised and homogenized with lysis buffer (Kurabo, Osaka, Japan), and then, the tissue lysate was centrifuged at 10,000 rpm. Plasma concentrations of interleukin (IL)-6, tumor necrosis factor (TNF)-α, creatinine, hyaluronic acid, endostatine, glutamate oxaloacetate transaminase (GOT), glutamic acid pyruvate transaminase (GPT), matrix metalloproteinase (MMP)-1, histamine, and angiopoitin 1 and 2 were measured using appropriate ELISA kits according to manufacturers’ instructions (IL-6, Enzo Life Sciences, Farmingdale, NY, USA; TNF-α, hyaluronic acid, and angiopoietin 2, R&D Systems, Minneapolis, MN, USA; MMP-1, MyBioSource, San Diego, CA, USA; histamine, Bertin Pharm, Montigny-le-Bretonneux, France; creatinine, Cayman, Ann Arbor, MI, USA; Endostatine, BioMedica, Vienna, Austria; angiopoietin 1, EIAAB Science, Wuhan, China; GOT and GPT, Wako, Osaka, Japan; ROS, Cell Biolabs Inc., San Diego, CA, USA). Optical density was measured using a microplate reader (Molecular Devices, Sunnyvale, CA, USA).

### 2.5. Western Blotting

The collected liver samples were homogenized with a lysis buffer and centrifuged at 1000 rpm at 4 °C for 20 min to collect the supernatant. SDS-PAGE was performed as previously described [14]. Protein was subsequently blocked for 1 h with 5% skim milk at room temperature. The membranes were washed with Tris-buffered saline–0.1% Tween 20 three times and were incubated at room temperature for 1 h with primary antibodies against Iba1 (marker of macrophage; 1:1000; Wako), chemokine receptor 7 (CCR7) (marker of M1 macrophage; 1:1000; Abcam, Cambridge, MA, USA), and β-actin as a loading control (1:5000; Sigma-Aldrich, St. Louis, MO, USA). Next, the membranes were washed and treated with horseradish peroxidase-conjugated secondary antibody (1:1000; Novex, Frederick, MD, USA), and the immune complexes were detected using ImmunoStar Zeta regent (Wako). The images were acquired using the Multi-Gauge software program (Fujifilm, Greenwood, SC, USA).

### 2.6. Statistical Analysis

All data are presented as mean ± standard deviation derived from five animals. Shown is a representative of 2 independent experiments. For comparisons between test groups, Tukey’s post hoc test or Steel–Dwass tests were applied, and differences with *p* < 0.05 were considered statistically significant.

## 3. Results

### 3.1. Effect of Aging on Body Weight, Blood Glucose Level, and Skin Condition in KK-Ay/Tajcl Mice

**Figure 1:**
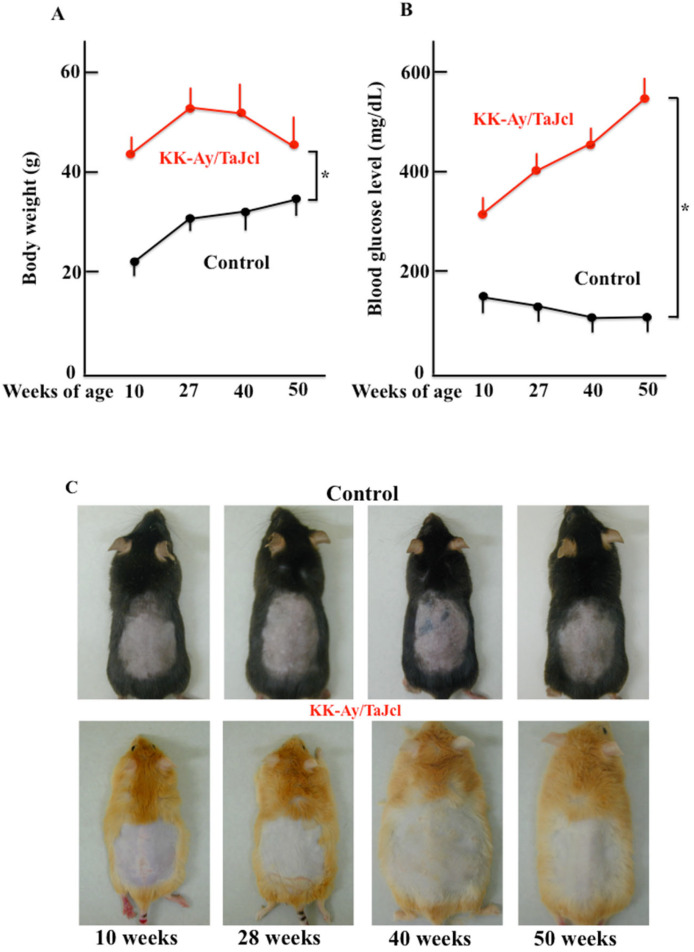
Effect of aging on body weight (**A**), blood glucose levels (**B**), and skin condition (**C**) in KK-Ay/TaJcl mice. Values are expressed as the mean ± SD of five animals. * *p* < 0.05. Control: C57BL/6j mice.

The body weight of control mice increased with age, but that of KK-Ay/Tajcl mice peaked at 27 w and decreased until 50 w (Figure 1A). Blood glucose levels did not change with age in the control group; however, KK-Ay/Tajcl mice showed diabetic values at 10 w of age, which increased over time (Figure 1B). Changes in skin condition were not macroscopically visible in either the control or KK-Ay/Tajcl mice groups over time (Figure 1C).

### 3.2. Effect of Aging on TEWL, Skin Hydration, Skin Thickness, and Mast Cell and Collagen Expression in KK-Ay/TaJcl Mice

**Figure 2:**
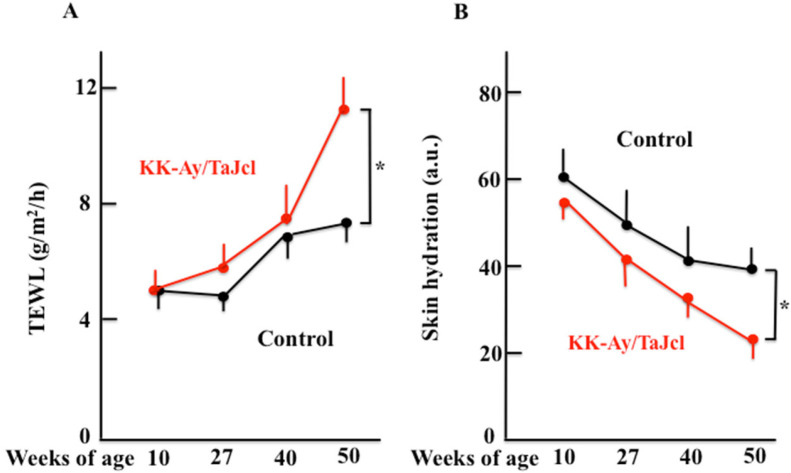

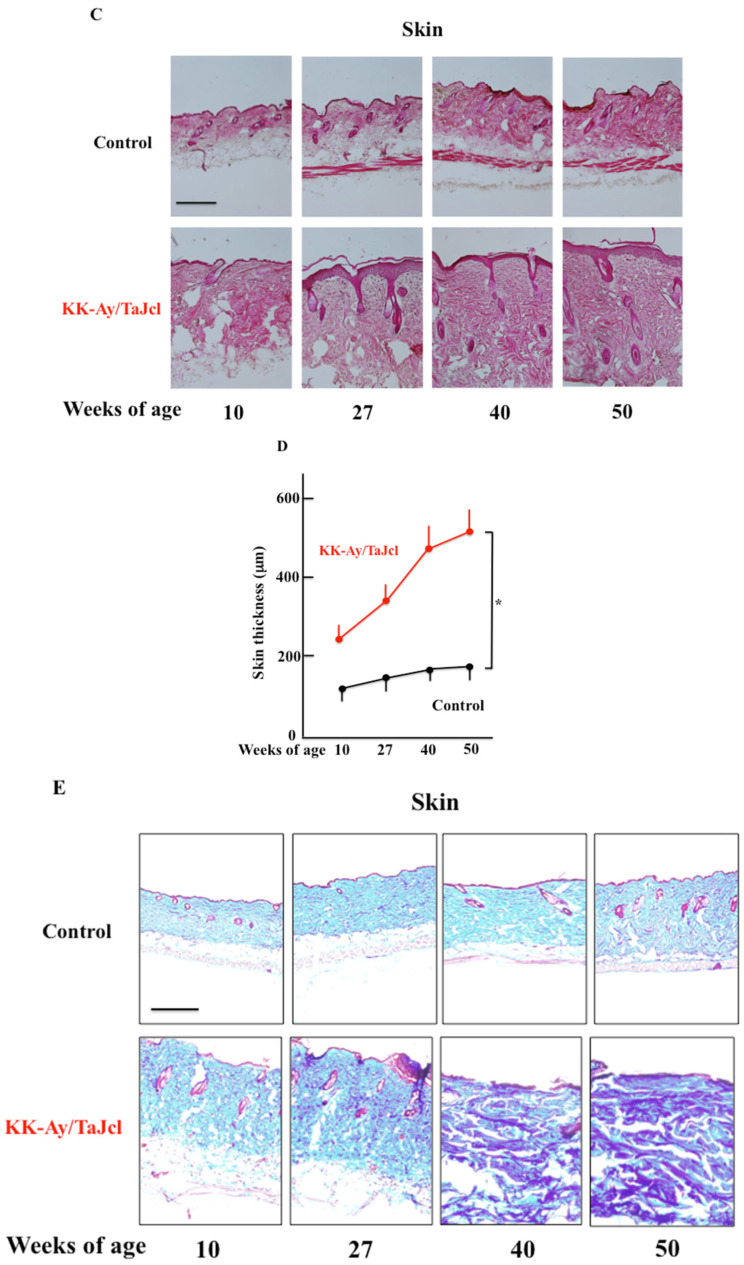
Effect of aging on transept water loss (**A**), skin hydration (**B**), and skin thickness (**D**) in KK-Ay/TaJcl mice. Histological analysis of skin sections was performed using hematoxylin–eosin (**C**) and Masson-trichrome (collagen stain) (**E**) staining. Values are expressed as the mean ± SD of five animals. * *p* < 0.05. Control: C57BL/6j mice. Scale bars (length unit display) = 100 μm.

To examine skin dryness, we first measured the TEWL (Figure 2A) and skin hydration (Figure 2B) levels of the stratum corneum in KK-Ay/TaJcl and control mice. We found that TEWL increased with age, while skin hydration decreased with age. In addition, both TEWL and skin hydration showed significant differences between KK-Ay/TaJcl mice and control mice in 50-w-old mice. When we measured skin thickness in these animals, no change was observed in the control group; however, skin became thicker with age in KK-Ay/TaJcl mice (Figure 2C,D). Moreover, the dermal expression of collagen decreased with age in KK-Ay/TaJcl mice when compared with the control (Figure 2E).

### 3.3. The Expression of Skin Mast Cell and Skin Histamine and Matrixmetalloproteinase-1 Expression in Aging KK-Ay/TaJcl Mice

**Figure 3:**
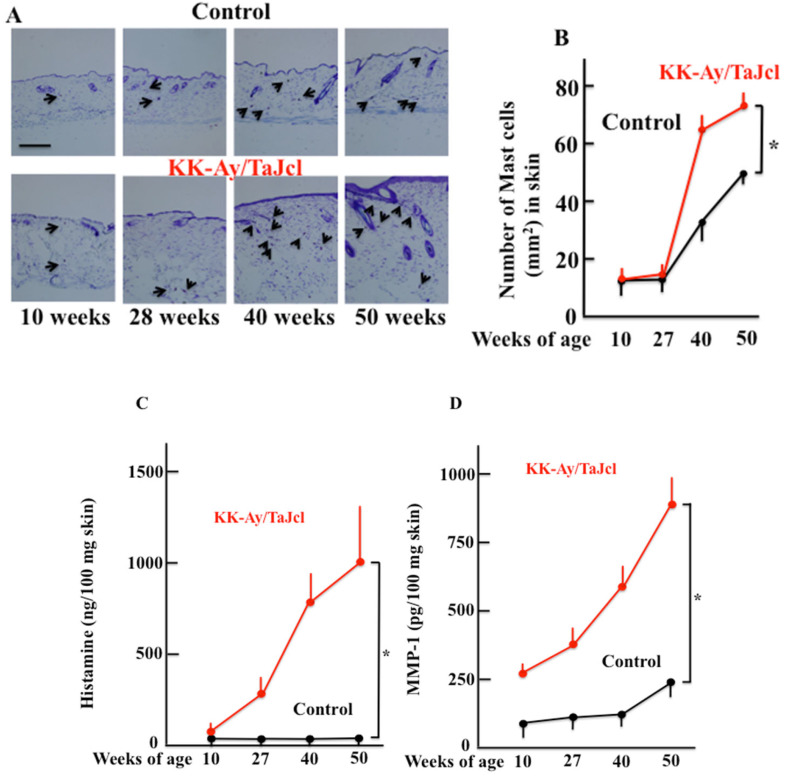
Effect of aging in KK-Ay/TaJcl mice on the expression of mast cells (**A**,**B**), and levels of histamine (**C**) and MMP-1 (**D**) in the dorsal skin. Arrows indicate mast cells stained with toluidine blue. The data show one representative experiment containing five animals. Scale bar = 100 μm. The dorsal skin levels of histamine and MMP-1 in mice were measured using ELISA. Values are expressed as mean ± SD derived from five animals. * *p* < 0.05. Control: C57BL/6j mice.

While the expression of skin mast cells increased from 27 w of age in both control and KK-Ay/TaJcl mice, there was a significant increase observed in KK-Ay/TaJcl mice when compared with that in the control (Figure 3A,B). Histamine secreted from mast cells both increased after 27 w in KK-Ay/TaJcl mice but did not change in control mice (Figure 3C), while matrix metalloproteinase-1 (MMP-1) increased after 10 w compared with that of control mice (Figure 3D).

### 3.4. Kidney Condition and Weight, and Blood Creatinine and Hyaluronic Acid Levels in Aging KK-Ay/Tajcl Mice

**Figure 4:**
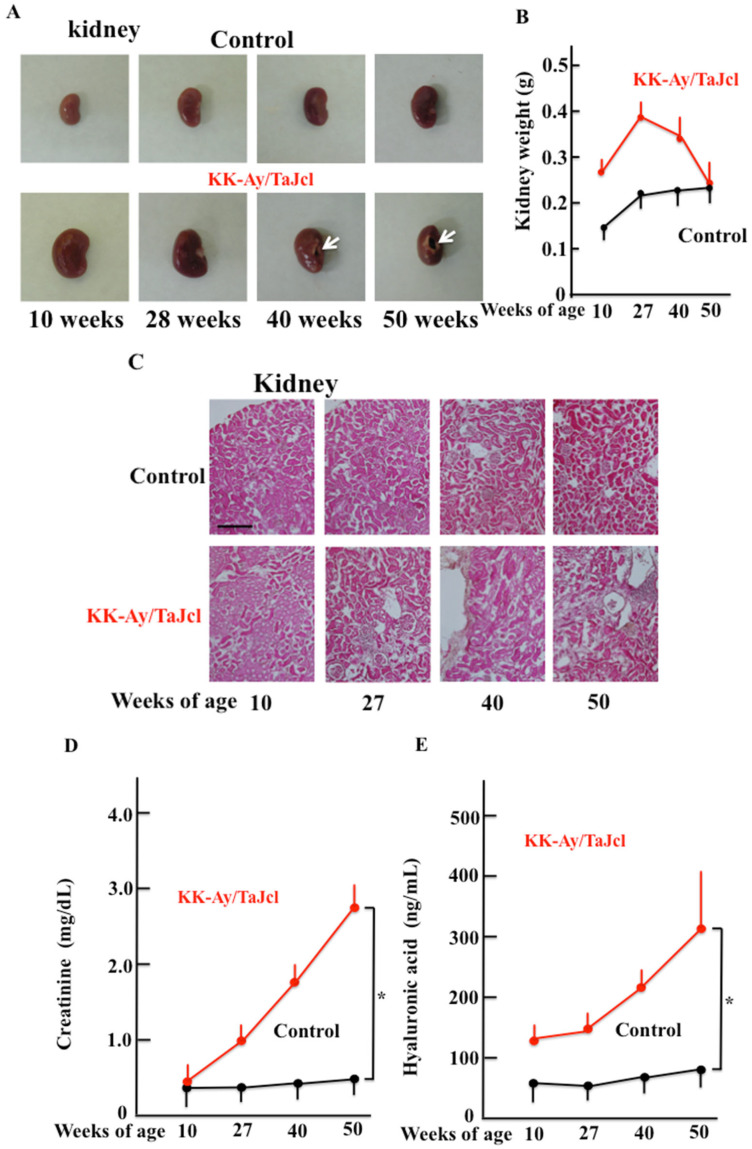
Effect of aging on kidney condition (**A**), kidney weight (**B**), histological analysis (hematoxylin–eosin staining) (**C**), and blood levels of creatinine (**D**) and hyaluronic acid (**E**) in the dorsal skin of KK-Ay/TaJcl mice. White arrows (**A**) indicate the site of kidney injury. The data show one representative experiment using five animals. Scale bar = 100 μm. Values are expressed as mean ± SD derived from five animals. * *p* < 0.05. Control: C57BL/6j mice.

The macroscopic observation of kidney samples from KK-Ay/TaJcl mice showed cavities in the kidney (arrows) and no change in the control with age (Figure 4A). The weight of the kidney did not change with age in the control mice, but KK-Ay/TaJcl mice increased in body weight at 27 w of age before it decreased and remained unchanged at 50 w of age (Figure 4B). The histological analysis of the kidneys showed that KK-Ay/TaJcl mice had interspersed tissue degeneration when compared with control animals (Figure 4C). In addition, the blood creatinine and hyaluronic acid levels, which are markers of kidney damage, increased with age in KK-Ay/TaJcl mice and not in control mice over time (Figure 4D,E).

### 3.5. Aging Effects IL-6, TNF-α and Endostatine Blood Levels and Kidney Angiopoietin 1 and 2 Levels in KK-Ay/Tajcl Mice

**Figure 5:**
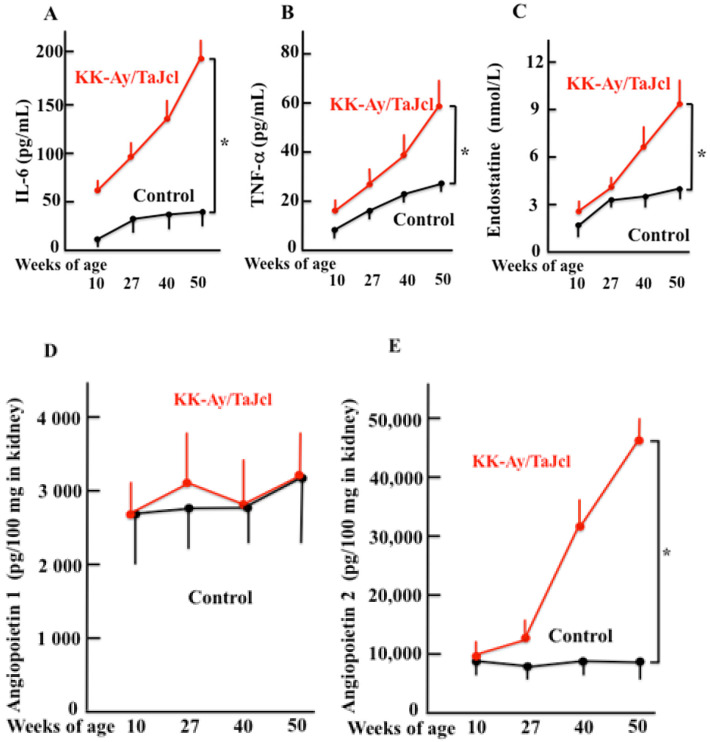
Effect of aging on the plasma of IL-6 (**A**), TNF-α (**B**), and endostatine (**C**) levels; and the kidney levels of angiopoietin 1 (**D**) and 2 (**E**) in KK-Ay/TaJcl mice. Plasma levels of TNF-α, IL-6, and IL-10, and the kidney levels of angiopoietin 1 and 2 in mice were measured with ELISA. Values are expressed as the mean ± SD derived from five animals. * *p* < 0.05. Control: C57BL/6j mice.

The blood levels of IL-6, TNF-α, and endostatin released by kidney damage increased with age, especially in KK-Ay/TaJcl mice when compared with the control (Figure 5A–C). The levels of angiopoietin 1 did not significantly change with age in either KK-Ay/TaJcl or control (Figure 5D); however, angiopoietin 2 significantly increased in KK-Ay/TaJcl mice after 27 w when compared with that in control mice (Figure 5E).

### 3.6. Liver Condition and Weight, and Glutamic Oxaloacetic Transaminase and Glutamic Pyruvic Transaminase Blood Levels in Response to Aging in KK-Ay/Tajcl Mice

**Figure 6:**
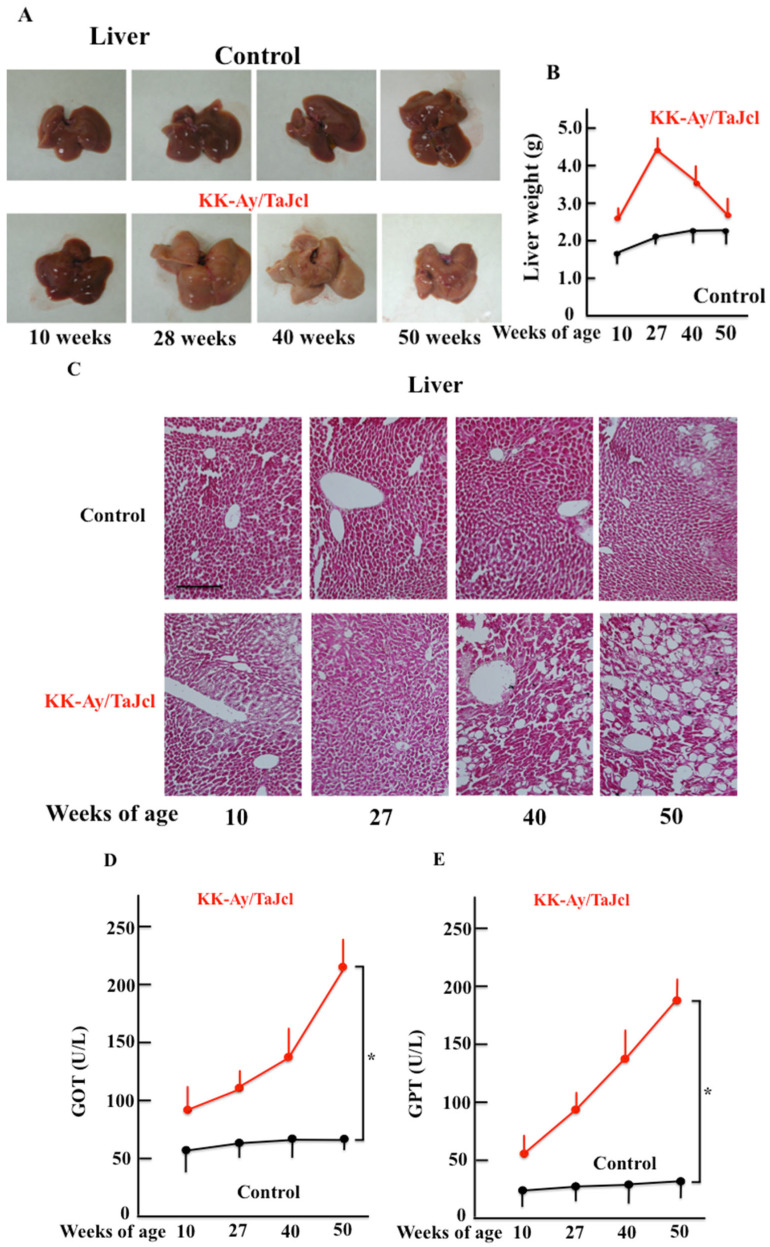
Effect of aging on liver condition (**A**), liver weight (**B**), and hematoxylin–eosin staining (**C**); and blood levels of GOT (**D**) and GPT (**E**) in the dorsal skin of KK-Ay/TaJcl mice. The data show one representative experiment performed on five animals. Scale bar = 100 μm. Values are expressed as mean ± SD derived from five animals. * *p* < 0.05. Control: C57BL/6j mice.

The macroscopic analysis of the livers of KK-Ay/TaJcl mice indicated a whitish coloration after 27 w and fatty liver development, while no changes were observed in control mice (Figure 6A). Furthermore, the liver weight peaked in the KK-Ay/TaJcl mice at 27 w and decreased thereafter, while it did not change in control mice (Figure 6B). The histological findings were consistent with liver degeneration and fat accumulation in KK-Ay/TaJcl mice (Figure 6C) and the indicators of liver damage, GOT and GPT, increased with age in KK-Ay/TaJcl mice when compared with the control (Figure 6D,E).

### 3.7. Effect of Aging on Reactive Oxygen Species and Ionized Calcium-Binding Adapter Molecule 1 and CC-Chemokine Receptor 7 Expression in KK-Ay/Tajcl Mice

**Figure 7:**
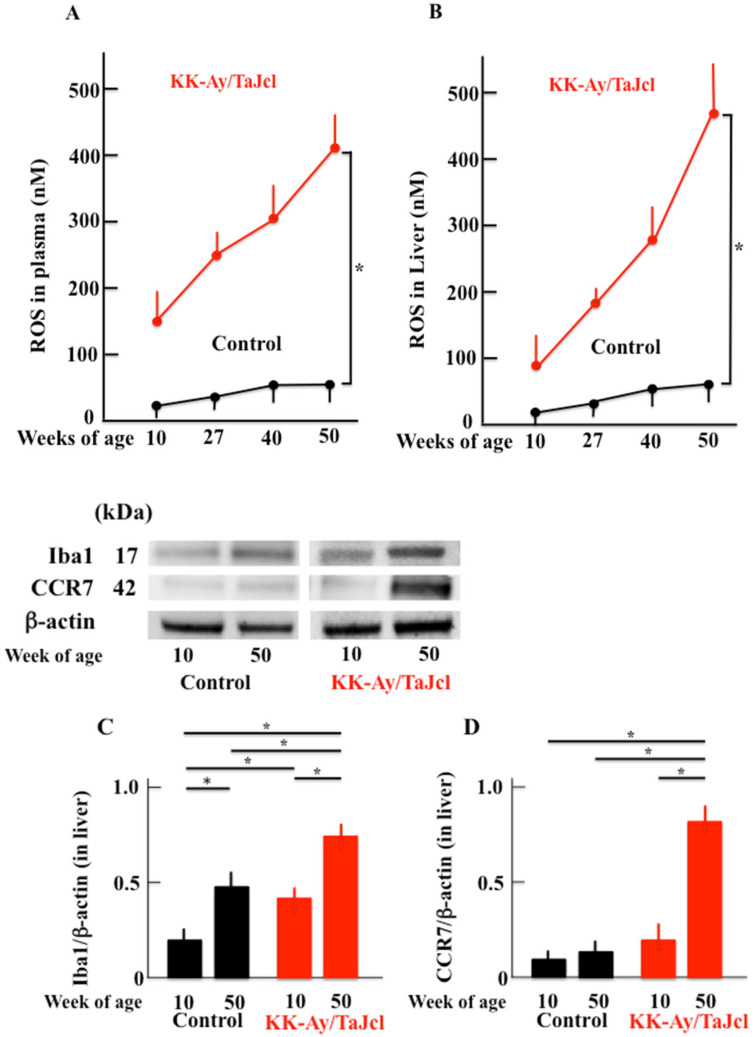

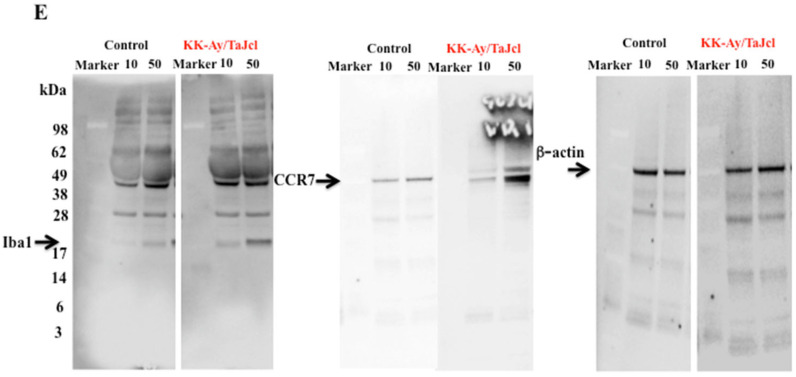
Effect of aging on ROS levels in the skin (**A**) and liver (**B**); and the expression of Iba1(**C**) and CCR7 (**D**) in KK-Ay/TaJcl mice compared with the control. Western blot diagram of Iba1, CCR7, and β-actin with molecular weight markers (**E**). We measured the levels of ROS using a commercial kit and Western blotting for the analysis of Iba1 and CCR7. Values are expressed as the mean ± SD derived from five animals. * *p* < 0.05. Control: C57BL/6j mice.

Reactive oxygen species (ROS) are expressed in fatty liver conditions and were increased in KK-Ay/Tajcl blood and liver samples with age, while no change was observed in the control mice throughout the test period (Figure 7A,B). The macrophage marker and ionized calcium-binding adapter molecule 1 (Iga1) increased between 10 and 50 w in both KK-Ay/Tajcl and control mice; however, KK-Ay/Tajcl mice showed significantly higher values when compared with the control (Figure 7C,E). The CC-chemokine receptor 7 (CCR7) is an index of M1 macrophages and showed a marked increase at 50 w in KK-Ay/Tajcl mice, while no change was observed in control mice (Figure 7D,E).

## 4. Discussion

In this study, the effects of the liver and kidney on skin dryness were investigated using the aging type 2 diabetes mouse model (KK-Ay/Tajcl). The dryness of the skin in KK-Ay/Tajcl mice worsened with age. We observed increased mast cells, histamine, and MMP-1 in the skin over time, while collagen type 1 decreased. In addition, kidney damage markers creatinine, endostatin, hyaluronic acid, and angiopoietin 2 increased with aging, indicating significant damage. Furthermore, fatty liver was observed in KK-Ay/Tajcl mice with aging, as well as the increased expression of the liver damage markers, GOT and GPT. In addition, ROS, M1 macrophages, and CCR7 increased.

We found that the aging in a diabetes mouse model induced severely dry skin. Aging KK-Ay/Tajcl mice at different time points showed significant renal damage; creatinine and hyaluronic acid increased, indicating kidney damage, as well as a large amount of inflammatory cytokine release [15,16]. Furthermore, an increase in renal angiopoietin 2 expression was also observed, which causes the microinflammation of blood vessels in chronic renal disease and promotes the extravasation of inflammatory cells [17,18]. Thus, the large number of inflammatory cytokines secreted by the kidney can activate mast cells [19,20] that release many bioactive substances, while histamine and MMP-1 cause the breakdown of skin collagen type 1 and exacerbate skin dryness [7,21].

As obesity progresses, fat accumulates in the liver and results in a fatty liver condition. Fatty acids increase oxidative and endoplasmic reticulum stress as the ability of hepatocytes to store triglycerides and oxidize fatty acids such as β-oxidation is exceeded. This increase in intracellular stress further promotes fat accumulation and causes a vicious circle that promotes hepatic fattening. Lipid mediators also strongly activate innate immunity via Toll-like receptors and induce liver and systemic inflammation [22], and the expression of ROS and inflammatory macrophages can increase [23,24,25]. These factors also affect the skin and can promote dryness. Furthermore, ROS and inflammatory cytokines released from the liver attack both the skin and kidneys; therefore, it is highly possible that kidney damage is further exacerbated, and the inflammatory cytokines released from the kidneys can damage the liver. In this way, the liver and kidney adversely affected each other and further increased skin damage.

**Figure 8:**
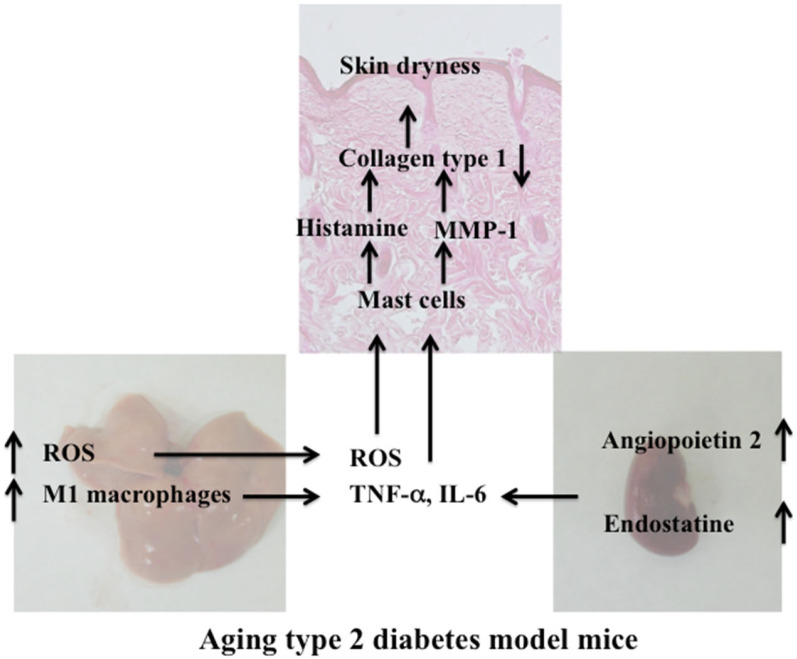
Skin/liver/kidney interactions that contribute to dry skin in aging KK-Ay/TaJcl mice.

The KK-Ay/Tajcl mice used in this experiment are an obese mouse strain that have a very high body fat content. The production of inflammatory adipocytokines such as TNF-α and IL-6 is enhanced in adipocytes, and the production of anti-inflammatory adipocytokines such as adiponectin is decreased [26]. In addition, macrophages that infiltrate obese adipose tissue are called M1 macrophages [26] and secrete inflammatory cytokines and adipokines such as TNF-α [27]. This inflammatory cytokine/adipokine response not only induces chronic inflammation, but also impairs insulin action [28]. In this study, blood TNF-α and IL-6 levels increased, but it is highly possible that they were secreted from adipose tissue in addition to the damaged kidneys; thus, it is necessary to investigate the role of adipocytes both in inflammation as well as kidney damage during obesity and aging. However, from the results of this study, weight loss occurred (i.e., decreased body fat mass), while blood glucose levels increased with aging in KK-Ay/Tajcl mice. From this, we hypothesized that the deterioration of skin dryness during aging was strongly affected by kidney and liver damage (Figure 8).

As diabetes progresses, dehydration occurs due to frequent urination and excessively high blood glucose levels change the collagen network, causing damage to blood vessels and stiffening the skin, which also causes dryness [29]. In this study, we measured the amount of urination and water intake (data not shown), both of which increase with age. The difference in urine output and water intake was the same as in young diabetic mice, suggesting that the possibility of dryness due to dehydration is low. On the other hand, skin stiffness due to vascular injury is well considered, and markers of vascular injury should be invested. Furthermore, this study concluded that the kidneys and liver are important for skin dryness because damage to the kidneys and liver increases with the progression of diabetes and correlates with increased skin dryness with aging. However, there is no direct evidence that the kidney and liver are interrelated and exacerbate dry skin due to diabetes, and further investigating is required.

## 5. Conclusions

In KK-Ay/Tajcl mice, skin dryness worsens with aging. In general, the physiological mechanisms of the body deteriorate with age, and various pathological conditions worsen. The occurrence of diabetes during aging leads to the further deterioration of skin, kidney, and liver functions. When these tissues are damaged, the secretion of ROS and inflammatory cytokines exacerbate skin dryness. Our results suggest skin/kidney/liver interaction that causes a negative feedback loop that contributes to the multiple pathologies observed during diabetes and aging. Based on these findings, it may be possible to create new drugs that target each organ in type 2 diabetes by further advancing this research. Furthermore, it may be possible to estimate the degree of kidney or liver damage by observing the skin condition. However, this study used mice, and it is necessary to confirm consistency with mice by conducting clinical trials on humans.

## Data Availability

Data are available within the article.
